# SCCMEC and SPA typing of meticillin resistance Staphylococcus aureus isolated from infections from Southern Poland

**DOI:** 10.1186/2047-2994-4-S1-P193

**Published:** 2015-06-16

**Authors:** A Chmielarczyk, M Pobiega, J Natkaniec, D Romaniszyn, I Myjak, K Malyszek, J Wojkowska-Mach

**Affiliations:** 1 Jagiellonian University Medical School, Kraków, Poland

## Introduction

The spa gene encodes protein A and is used for typing of methicillin-resistant *Staphylococcus aureus*, MRSA, such as the mec operon carried by staphylococcal cassette chromosome (SCCmec).

## Objectives

The aim of this study was the molecular typing of MRSA isolated from different forms of infections for epidemiological purposes using spa typing method and SCCmec classification.

## Methods

A total of 90 MRSA isolates coming from eight hospitals from southern Poland were tested. Isolates originated from: bloodstream and respiratory tract infections (36), surgical site infections (30), chronic wounds (24). S *pa* typing was performed as described previously [1], using the spa typing website (http://www.spaserver.ridom.de/). Staphylococcal cassette chromosome *mec* (SCC *mec*) typing was performed as described previously [2].

## Results

The majority of MRSA strains were of SCCmec type II (42.2%) or SCCmec IV (21.1%). Eleven strains was marked as SCCmec III (12.2%), 8 as SCCmec V (8.9%), 4 as SCCmec I (4.4%) and one as SCCmec VI.

The spa type t003 was the most frequently observed (37.8% of strains), then t138 (14.4%), t008 and t037 and t041 (4.4%),

SCC *mec* type II and spa-t003 together were characteristic for 25% of the chronic wounds and 27% in SSI, but 39% in invasive infections.

SCCmec III and t138 occurred in 10% of the strains from two hospitals, SCCmecIV and t003 occurred in 6.7% strains from two hospitals.

## Conclusion

Epidemiological and molecular studies of MRSA isolates allowed to detail insight into the problem of staphylococcal infections. Those methods are less time-consuming than PFGE and give the opportunity for the observation of the current situation and epidemiological trends for resistant strains on the level of ward/hospital or even whole region (supported by a grant DEC-2011/03/B/NZ7/01911).

## Disclosure of interest

None declared.
